# Biochemistry-informed design selects potent siRNAs against SARS-CoV-2

**DOI:** 10.1080/15476286.2023.2217400

**Published:** 2023-06-04

**Authors:** Élisabeth Houbron, Sophie Mockly, Sophia Rafasse, Nathalie Gros, Delphine Muriaux, Hervé Seitz

**Affiliations:** a Institut de Génétique Humaine, UMR 9002 CNRS and University of Montpellier, Montpellier, France; b Institut de Recherches Cliniques de Montréal (IRCM), Montréal, QC, Canada; c CEMIPAI UAR 3725 CNRS and University of Montpellier, Montpellier, France; d IRIM, UMR 9004 CNRS and University of Montpellier, Montpellier, France

**Keywords:** siRNA, RNAi, SARS-CoV-2, siRNA design, RNA accessibility

## Abstract

RNA interference (RNAi) offers an efficient way to repress genes of interest, and it is widely used in research settings. Clinical applications emerged more recently, with 5 approved siRNAs (the RNA guides of the RNAi effector complex) against human diseases. The development of siRNAs against the SARS-CoV-2 virus could therefore provide the basis of novel COVID-19 treatments, while being easily adaptable to future variants or to other, unrelated viruses. Because the biochemistry of RNAi is very precisely described, it is now possible to design siRNAs with high predicted activity and specificity using only computational tools. While previous siRNA design algorithms tended to rely on simplistic strategies (raising fully complementary siRNAs against targets of interest), our approach uses the most up-to-date mechanistic description of RNAi to allow mismatches at tolerable positions and to force them at beneficial positions, while optimizing siRNA duplex asymmetry. Our pipeline proposes 8 siRNAs against SARS-CoV-2, and *ex vivo* assessment confirms the high antiviral activity of 6 out of 8 siRNAs, also achieving excellent variant coverage (with several 3-siRNA combinations recognizing each correctly-sequenced variant as of September2022). Our approach is easily generalizable to other viruses as long as avariant genome database is available. With siRNA delivery procedures being currently improved, RNAi could therefore become an efficient and versatile antiviral therapeutic strategy.

## Introduction

RNA interference (‘RNAi’) is an efficient repressive mechanism broadly conserved among eukaryotes. While there exists adiversity of clade-specific features [1], the core mechanism of RNAi always involves small RNA guides (‘siRNAs’) loaded on aprotein of the AGO subfamily of the Argonaute family [2]. The resulting ribonucleoprotein complex (the ‘RISC’ complex) then binds target RNAs exhibiting sufficient sequence complementarity, and cleaves them endonucleolytically between the nucleotides facing guide nucleotides 10 and 11 [3–7]. Mismatches in the vicinity of the scissile phosphate tend to inhibit the cleavage reaction [4,8–11].

siRNAs are double-stranded (with each strand being ≈ 21 nt long). After loading on the AGO protein, one strand (the ‘passenger strand’) is discarded while the other one (the ‘guide strand’) remains stably associated with AGO. Mature RISC therefore contains asingle-stranded small RNA, which is available for base-pairing with target RNAs [12]. Among the four existing AGO’s in mammals, only one (named ‘AGO2’) possesses acompetent catalytic site for robust cleavage activity [13–16].

Besides RNAi, other related pathways also implicate small RNA-loaded Argonaute proteins. In particular, the microRNA (‘miRNA’) pathway, where the small guide RNA follows adistinctive biogenesis route before being loaded on an AGO protein [1]. In bilaterian animals, miRNAs typically recognize their targets by imperfect sequence complementarity: in general, the target exhibits aperfect match to the miRNA ‘seed’ (nt 2–7), but mismatches are well tolerated at most other positions [17]. Just like siRNAs, miRNAs can guide target cleavage if sequence complementarity requirements are met [18], but this is very rare in bilaterian animals [19–22]. If conditions are not met for target cleavage (either because of mismatches close to the scissile phosphate, or because the AGO protein does not possess acatalytic site for cleavage), the target RNA is still repressed, but less efficiently, by various mechanisms (including exonucleolytic degradation and, for mRNAs: translational inhibition [23]).

The efficiency and specificity of RNAi make it apowerful tool for targeted gene repression in laboratory settings. It has also quickly been proposed to serve as abasis for antiviral therapy [24] but clinical applications have been slower, with only 5 approved therapeutic siRNAs so far, none of them targeting avirus [25–29]. Obstacles to the development of therapeutic siRNAs include the poor stability of RNA in serum, and the tendency for injected siRNAs to concentrate primarily in the liver. Efforts for the development of these five approved drugs resulted in the discovery of chemical modifications which greatly stabilize siRNAs *invivo* while not compromising too much their activity. Regarding pulmonary diseases, the biodistribution issue may also be circumvented by the development of inhalation-based procedures [30].

**Figure 1.**
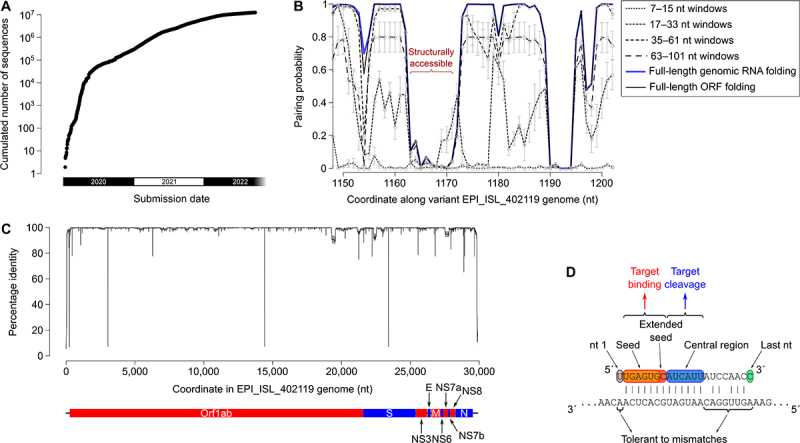
Criteria for siRNA design.

Notes: a. Cumulated number of SARS-CoV-2 variant sequences from human donors at GISAID as afunction of time. b. Predicted structural accessibility of nt 1150–1200 in viral variant #EPI_ISL_402119 (a.k.a. BetaCoV/Wuhan/IVDC-HB-01/2019). x axis: genomic coordinate. y axis: nucleotide pairing probability in various sequence contexts (full-length genomic RNA folding, full-length ORF folding, and various sliding window size ranges). Gray circles represent the mean pairing probability across window sizes and gray error bars represent the standard error. The red curly bracket indicates astructurally accessible region. c. Nucleotide conservation along EPI_ISL_402119 genome sequence. 100 non-overlapping sets of 100 variants each were randomly picked among the 311,994 correctly-sequenced viral variants from human donors in GISAID release dated Jan.8, 2021, then each of these sets was aligned with the EPI_ISL_402119 sequence. Identity to EPI_ISL_402119 was scored for each nucleotide in that reference sequence; the black curve represents the mean, and the gray shading represents the mean ± standard deviation, across all 100 random sets. Annotated ORFs in the EPI_ISL_402119 genome are represented under the graph (red and blue bars). d. Functional modules in asiRNA guide strand sequence (top strand) for the recognition and cleavage of atarget RNA (bottom strand). Guide nt 2–7 constitute the ‘seed‘ and nt 2–8 constitute the ‘extended seed‘ [17,33]. Guide nt 9–13 (and 9–14 for some siRNA sequences) need to be perfectly paired to the target for optimal cleavage rate according to Cleave’n-Seq experiments [7]. Other positions are more tolerant to mismatches without compromising target binding or cleavage. The last nucleotide is preferentially unpaired (respectively to its target) in order to accelerate product release and reduce the risk of TDMD-mediated siRNA destabilization or guide strand unloading [7,11,34].

The coronavirus disease 2019 (COVID-19) outbreak started during the winter 2019–2020 and it was initially detected in Wuhan (China) [31]. The causative viral agent, the severe acute respiratory syndrome coronavirus 2 (SARS-CoV-2), was quickly identified and its genome was sequenced from patient samples and distributed via the GISAID repository [32]. Hundreds of viral variants from individual donors were available in the first few weeks of the pandemic (see Figure1a). Because the biochemistry of RNAi is well known, it is possible to design siRNA sequences with predicted high efficacy– and because so many sequenced viral variants were quickly available, it is possible to choose siRNA sequence sets which should be active against every known variant. Here we describe our bioinformatics approach for the design of siRNAs against SARS-CoV-2, and verification of their activity in asimple, SARS-CoV-2 infected cultured cell-based model *ex vivo*. Acomparison with previously published works shows that our purely computational design yields ahigh proportion of efficient siRNAs against SARS-CoV-2 that were validated *ex vivo*. Once delivery methods have been optimized for *invivo* administration, antiviral siRNAs could therefore offer aquick response, easily designed and easily reprogrammable against novel variants, or other viruses in the case of future viral pandemics.

## Materials and methods

### Computational analyses

Viral variant sequences were downloaded from the GISAID database (https://gisaid.org/). Conserved regions were identified from **clustalw**-generated alignments of correctly sequenced variant sequences (at least 26 kb long, no more than 5% ‘N’ ambiguities). RNA secondary structures in viral RNAs were predicted using **RNAfold** on areference variant sequence (#EPI_ISL_402119).

Scripts, raw, intermediate and final data files are available at https://github.com/HKeyHKey/Houbron_et_al_2023 and at https://www.igh.cnrs.fr/en/research/departments/genetics-development/systemic-impact-of-small-regulatory-rnas#programmes-informatiques/.

### Cell culture, transfection and assessment of siRNA cytotoxicity

Vero E6 cells (ECACC batch #15A033) were grown in DMEM (with 10% FBS, 1% L-glutamine, 1% penicillin+streptomycin, no glutamax; Gibco) at 37°C under 5% CO_2_. siRNAs were transfected using Lipofectamine RNAimax (Invitrogen) according to the manufacturer’s protocol, 24 h after seeding 96 well-plates with $3.75\times 10^{3}$ cells per well (for the siRNA cytotoxicity assay) or $3\times 10^{4}$ cells per well (for antiviral assessment by RT-qPCR or cytopathic effect measurement). siRNA cytotoxicity was assessed 72 h post-transfection using the CellTiter Glo kit (Promega). For A549-hACE2 cells (strain described in [35,36]), protocols were identical except that the cells were grown in RPMI with L-glutamine (with 10% FBS, 1% penicillin+streptomycin; Corning), transfected with Lipofectamine 3000 (Invitrogen) according to the manufacturer’s protocol, and seeded at $2.5\times 10^{3}$ cells per well for the siRNA cytotoxicity assay, and $2\times 10^{4}$ cells per well for the antiviral activity assay.

### Viral infection and assessment of antiviral activity

Six hours after siRNA transfection, the medium was removed, cells were rinsed with PBS, then incubated in fresh DMEM (for Vero E6) or RPMI (for A549-hACE2) medium. Onehour later, SARS-CoV-2 (strain #2020-A0093534 isolated by CPP Île de France III and provided by Collection resource CRB of the Montpellier hospital) was inoculated at MOI = 0.01. Cytopathic effect was assessed using the Viral ToxGlo Assay kit (Promega) 72 h after infection. For viral RNA quantification: cells were rinsed with PBS, lysed with the Luna Cell Ready Lysis Module (New England BioLabs): in a96 well plate, cells were lysed in 40 μLlysis buffer per well (10 min incubation at 37°C, then 5 μLStop mix was added to each well), then lysates were transferred to anew plate and frozen at −80°C. 1 μLlysate was used as atemplate for the Luna Universal One-Step RT-qPCR Kit (New England Biolabs) on aLight Cycler 480 (Roche). Viral RNA (mRNA for gene E) was amplified with primers ACAGGTACGTTAATAGTTAATAGCGT and ATATTGCAGCAGTACGCACACA; for normalization, cellular GAPDH mRNA was amplified with primers GCTCACTGGCATGGCCTTCCGTG and TGGAGGAGTGGGTGTCGCTGTTG.

As apositive antiviral control, Remdesivir was added to the cell medium at 10 μMafter viral infection.

### Ethics

Ethical approval was obtained from the cell culture room’s institutional review board and ethics committee (protocol number 2010-A01406-33) as is classically done in such studies.

## Results

### Rationale for siRNA design

Biochemical determinants of siRNA activity are now very well described, therefore theoretically offering the possibility to design efficient siRNAs with apure computational approach. Because SARS-CoV-2 is apositive-strand RNA virus, siRNAs raised against genomic RNA can also target viral mRNAs, while siRNAs against the negative strand would only target short-lived replication intermediates, resulting in alower efficiency [37]. We therefore applied the following criteria for the design of positive strand-targeting siRNAs against SARS-CoV-2:

- Target sites are poorly accessible to siRNAs if they can fold on themselves, forming stable intramolecular stems [38]. Target secondary structure can be predicted by conceptual folding of the full-length genomic RNA, full-length mRNAs (here approximated by their ORF’s), or by shorter sliding windows (short-range interactions are expected to re-form rapidly after ribosome scanning). We therefore selected siRNA candidates targeting viral regions which are predicted to be accessible according to each of these metrics (see Figure 1b).
- Viral genomes tend to mutate very quickly, and it can be anticipated that, in the long-term, an siRNA treatment would promote the appearance of resistant variants (where the siRNA target site has been mutated). We therefore selected siRNA candidates which can target the most conserved viral regions: not only they should be active against already-existing variants, but likely also against future variants (strong sequence conservation in the viral genome marks functionally important segments, which could not mutate without compromising viral spread) (see Figure 1c).
- If imperfect base-pairing between the siRNA guide strand and the target cannot be avoided (which is the case when designing siRNAs against alarge diversity of viral variants), mismatches may be harmlessly introduced at positions 1, 15, 17, 18, 19, 20 or 21 of the guide RNA [7,39,40]. Mismatches at other positions often reduce binding affinity of RISC for its target, or the catalytic rate of the cleavage reaction— sometimes in asequence-dependent manner. The first nucleotide of the siRNA guide strand does not need to be complementary to the facing target nucleotide: it is anyway unpaired in the RISC complex, even when the facing nucleotide is complementary [41]. We therefore selected 13-mer target regions (to be targeted by siRNA guide nt 2–14) which are conserved among viral variants (see Figure 1d).
- Human AGO2 binds preferentially small RNAs with a5´ uridine or a5´ adenosine [42]. And more precisely, natural human miRNAs frequently have a5´ uridine [43], which may be due to an intrinsically higher affinity of the AGO protein or its loading machinery. We therefore introduced a5´ uridine in each siRNA candidate.
- In addition to the intended target, introduced siRNAs are likely to bind additional mRNAs (‘off-targets’): these are human endogenous mRNAs with fortuitous binding sites for the siRNA. If there are many off-targets, the siRNA is likely to be partially titrated, hence less efficient [44]. And because off-targets are (moderately) repressed by the siRNA, they could trigger unwanted secondary effects [45]. The most efficient binding sites (‘7mers’ and ‘8mers’ [17]) are so short that it is impossible to avoid off-targets (some human mRNAs will unavoidably contain perfect seed matches in their 3´ UTR). But it is possible to minimize the number of off-targets, and to minimize the number of off-targets whose modest down-regulation is most susceptible to trigger phenotypic consequences in humans (here approximated by acurated list of human haplo-insufficient genes [46]).
- In an siRNA duplex, differential base-pairing stability on the two ends of the duplex principally determines the identity of the guide strand [47–49]. Fraying the 5´-most nucleotide of the intended guide strand, and favoring candidate siRNAs with intrinsic asymmetry (high AU-content at the 5´ end of the intended guide strand, high GC-content at the 5´ end of the intended passenger strand) therefore improves siRNA efficiency.
- Once the RISC complex is matured, it is more stable if the guide strand contains a5´ phosphate [15]. Functional asymmetry of the duplex can therefore be further improved by phophorylating the 5´ end of the intended guide strand, while the 5´ end of the intended passenger strand is unphosphorylated [50].

**Figure 2.**
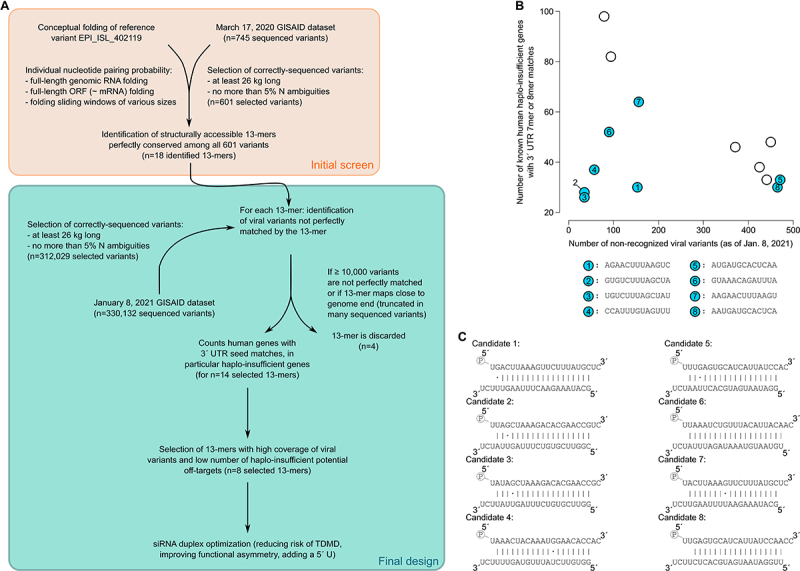
siRNA selection.

Notes: a. Workflow for siRNA design. An initial screen (orange frame) identified alist of 18 candidate 13-mer structurally-accessible target sites conserved among sequenced variants as of March17, 2020. Final siRNA design screened these 18 candidates for their conservation in an updated variant list (as of January8, 2021) and ranked them by their number of un-recognized viral variants and their number of predicted haplo-insufficient off-targets. b. Final choice of 8 target 13-mers. x-axis: number of non-recognized viral variants (*i.e*.: viral variants without aperfect 13-mer match not due to sequence ambiguity) out of 312,029 variants in total. y-axis: number of haplo-insufficient predicted human off-targets (*i.e*.: bearing a7mer or 8mer site for the siRNA seed in their 3´ UTR). Eight 13-mers with low numbers of missed viral variants and low numbers of predicted off-targets were selected and siRNAs were raised against each (these 13-mers are colored in blue, their sequences are given under the graph). c. Optimized siRNA duplexes targeting the 8 selected 13-mer targets. In each duplex, the intended guide strand is at the top and the intended passenger strand is at the bottom.

Our siRNA design workflow is presented in Figure 2a. It resulted in the selection of 8 target 13-mers whose seed match is structurally accessible according to every metrics, which are perfectly conserved in >99.8% correctly-sequenced viral variants, and which tend to minimize the number of haplo-insufficient predicted off-targets (see Figure 2b). Even though our method was applied to the whole viral genome, each of these 8 candidate target sites is located in the Orf1ab coding sequence of the viral genome (see below, section ‘Comparison to previously-published siRNAs and recent viral variants’). This is probably due to the fact that Orf1ab is the longest gene in SARS-CoV-2 (accounting for more than two-thirds of the total genome length), as well as to the presence of long stretches of highly constant regions coding for viral enzymes expressed from that gene. siRNA duplexes against these 8 target sites are presented in Figure 2c: they were optimized for their functional asymmetry (with the intended guide strand being 5´-phosphorylated, and having less stably paired 5´ nucleotides than the intended passenger strand), and the 3´-most nucleotide of their intended guide strand cannot pair to the facing nucleotide in the viral RNA target. Fraying the guide’s 3´ end when paired to its target indeed accelerates product release and reduces the risk of guide strand destabilization by TDMD (target-directed microRNA degradation) or by AGO unloading [7,11,34].

#### *Ex vivo* assessment against SARS-CoV-2

**Figure 3.**
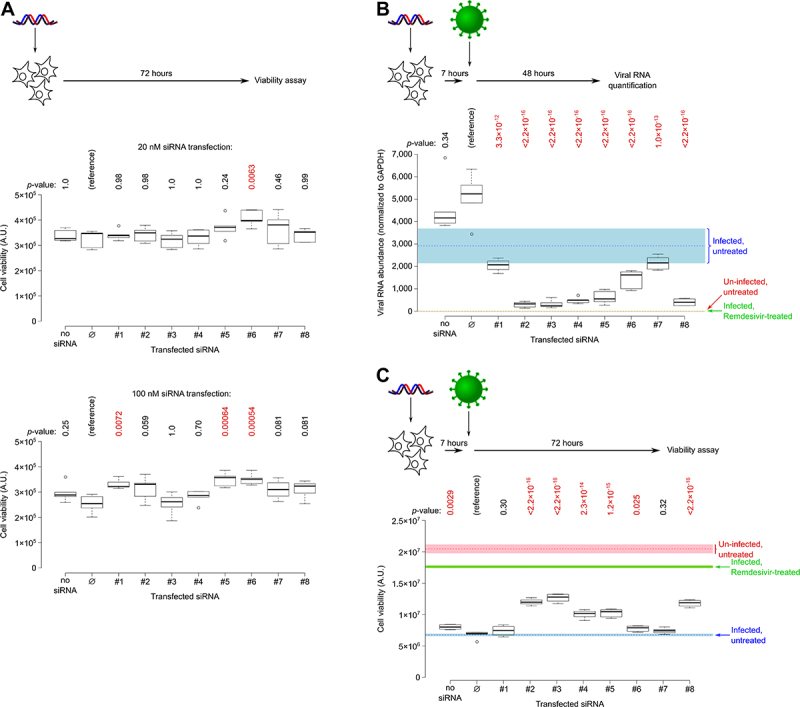
*Ex vivo* assessment of siRNA cytotoxicity and antiviral activity.

Notes: a. Cell viability was measured 72 hafter siRNA transfection (5 biological replicates). band c: Cells were transfected with 20 nM siRNA and infected 7 hlater (2 replicates for ‘Un-infected, untreated‘ and for ‘Infected, Remdesivir-treated‘, an antiviral positive control; 4 replicates for ‘Infected, untreated‘; 6 replicates for other conditions). Colored rectangles indicate the mean ± standard error across replicates for infection and treatment controls. b. Viral RNA was quantified by RT-qPCR 48 hafter infection, using endogenous GAPDH mRNA for normalization. c. Cell viability was measured 72 hafter infection (see Supplementary Figure S2 for additional siRNA concentrations). a, band c: Controls: ‘no siRNA‘ were transfected with empty Lipofectamine; ‘∅‘ were transfected with acontrol siRNA (designed to minimize its number of seed matches in mammalian genomes, while not having any 13-mer match to any sequenced SARS-CoV-2 variant as of Jan.8, 2021). Significance was assessed using Dunnett’s test, with the ‘∅‘ siRNA as areference; p-values are indicated above each graph, with p-values lower than 0.05 being shown in red.

The eight candidate siRNAs were synthesized and transfected in Vero E6 cells for cytotoxicity assessment. Vero E6 is afoetal *Cercopithecus aethiops* monkey kidney cell line particularly permissive to replication of SARS-CoV-2, and where the virus causes astrong cytopathic effect [51]. In the conditions tested, none of the siRNAs triggered significant cell death 72 h after transfection. Three candidates (siRNAs #1, 5 and6) actually increased cell proliferation at high concentration (100 nM), with one of them (siRNA #6) also significantly increasing proliferation at 20 nM (see Figure 3a), which remains unexplained. We also verified the absence of siRNA-induced lethality in ahuman cell line (the lung epithelium-derived A549 line expressing astable hACE2 transgene to make it susceptible to SARS-CoV-2 infection [52]). In this human-derived cell line too, none of the 8 siRNAs induced cell death (see Supplementary Figure S1A).

Antiviral activity was then evaluated by transfecting 20 nM siRNAs in Vero E6 cells, then infecting the cells with SARS-CoV-2 seven hours post-transfection. The virus is not washed out letting the infection to propagate for 3 days. Viral RNA contained in the cells was quantified by RT-qPCR on gene E48 h after infection, and the cytopathic effect (CPE) was quantified by measuring cell viability 72 h after infection. These two independent measurements gave similar conclusions, with siRNAs #1 and 7 showing no clear antiviral activity, siRNA #6 showing amoderate activity, while siRNAs #2, 3, 4, 5 and 8 showed both astrong reduction in viral RNA titre and in CPE (see Figure 3b and c). Cytopathic effect was also assessed after transfection of various siRNA concentrations, followed by viral infection: results are very similar to the 20 nM siRNA condition (see Supplementary Figure S2). In the A549-hACE2 cell line too, the same 6 siRNAs exhibited high anti-viral activity as judged by RT-qPCR on gene E(see Supplementary Figure S1B).

A combination of two siRNAs (siRNAs #3 and8) or of three siRNAs (siRNAs #3, 4 and8) did not trigger significant cell death when transfected at atotal concentration of 20 or 100 nM, either in Vero E6 or in A549-hACE2 cells (see Supplementary Figure S3Aand B). Their anti-viral activity in Vero E6 cells was moderate (≈40% reduction in viral RNA) while it was similar to that of individual siRNAs in A549-hACE2 cells (92–95% reduction): see Supplementary Figure S3Cand D.

### Comparison to previously-published siRNAs and recent viral variants

**Figure 4.**
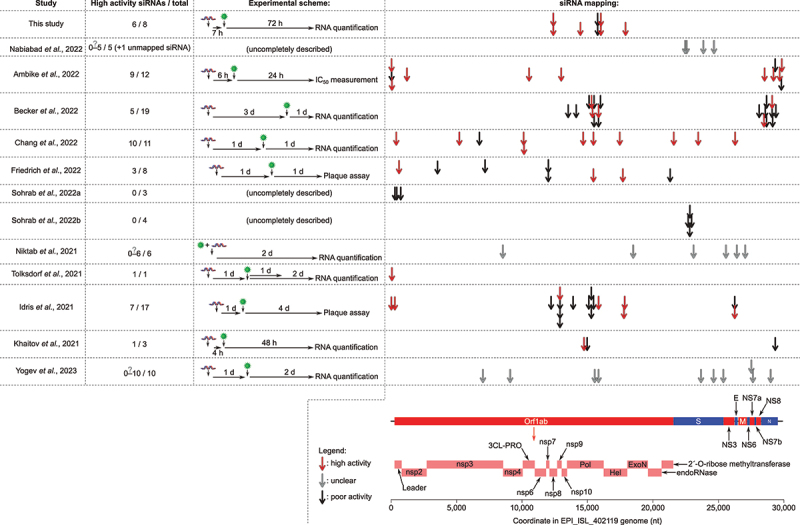
Comparison with previous anti-SARS-CoV-2 siRNA design attempts.

Notes: Each arrow represents the target site for apublished siRNA (red arrows indicate siRNAs with areported high activity; gray arrows indicate siRNAs with unclear activity; black arrows indicate siRNAs with poor activity). ‘High activity‘ was defined as asignificant (p< 0.05) repression of viral RNA accumulation by ≥50% in infected cultured cells [53,55,57–59,61,62] (and this study), or significant repression of viral plaque count by ≥50% [54,60], or ameasured IC_50_ for cell infection inhibition being ≤10 nM [37]. siRNAs have unclear activity when they were assessed in asiRNA mixture rather than individually [56], when they were combined with an anti-hACE2 aptamer before administration to cultured cells [63], or when they were only tested at a100 nM concentration [58] (gray arrows; also denoted by aquestion mark for the number of highly active siRNAs in column ‘High activity siRNAs/total‘). SARS-CoV-2 ORF coordinates are from GenBank ACC # OP278726.1; mature proteins generated from the Orf1ab polyprotein are indicated by pink rectangles.

Several studies have already described the design and experimental evaluation of siRNAs against SARS-CoV-2, with some siRNAs being more active than others [37,53–63]. Comparing success rates among diverse studies is not straightforward because of methodological differences between studies. To make the comparison as fair and uniform as possible, we compiled the results of *ex vivo* assessment of siRNAs against SARS-CoV-2 and flagged ‘high activity’ those siRNAs with asignificant repressive effect on viral RNA accumulation or viral plaque forming units exceeding 50% in the tested conditions (selecting conditions with siRNA concentrations close to 10 nM and post-transfection incubation close to 48 h whenever possible). The proportion of designed siRNAs with high activity ranges from 0 [57,59] to 10/11 [61] and 1/1 [55] (see Figure 4; note that siRNAs designed by [58] could not be analysed because they were tested *ex vivo* against SARS-CoV-2 only at a100 nM concentration). Our approach, yielding 6/8 siRNAs with high activity, proved efficient in designing potent siRNAs with apure bioinformatics approach, without requiring high-throughput experimental screening.

**Figure 5.**
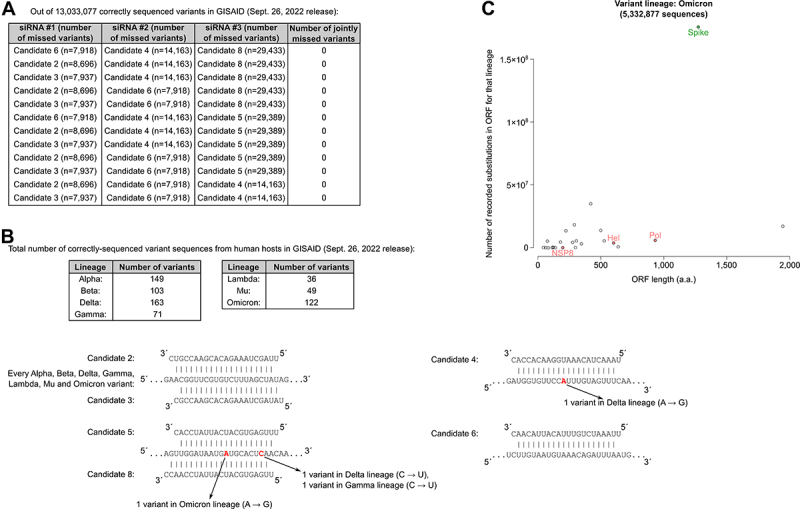
Recognition of newly emerged viral variants.

Notes: a. Among the 20 possible 3-siRNA combinations among our 6 siRNAs with high activity, 12 combinations do not miss any of the correctly sequenced variants (as of September26, 2022), excluding variants that were not matched perfectly because of sequence ambiguities. A‘missed variant‘ is avariant not exhibiting aperfect 13-mer match to nt 2–14 of the siRNA guide strand. b. 7 variant lineages of concern or of interest (Alpha to Omicron) are recorded in the latest GISAID release at the time of writing, with various numbers of deposited sequences (see tables at the top). Each of these variant sequences is perfectly recognized by our siRNA Candidates #2, 3 and 6 and almost all of them are recognized by Candidates #4, 5 and 8 (see predicted RNA duplexes at the bottom). c. Number of recorded amino acid substitutions in Omicron lineage variants in GISAID (Sept. 26, 2022 release) for each viral protein (y axis) as afunction of their length (x axis). The Spike protein is highlighted in green, while the three proteins whose gene is targeted by our siRNAs are shown in pink. The total number of Omicron lineage variants in that GISAID release is given above the graph. See Supplementary Figure S4 for other variant lineages of concern or of interest.

Our siRNA optimization procedure was performed on the 8January2021 GISAID variant collection (330,132 sequenced variants, including 311,994 correctly sequenced variants from human donors). In the course of the study, many additional variant sequences (including variants of concern) have been deposited at GISAID: at the time of writing, 13,243,751 variant sequences were available, including 13,033,077 correctly sequenced variants collected from human donors (26September2022 GISAID dataset). Even in this updated variant list, and after excluding unrecognized variants because of sequence ambiguities, our 6 siRNAs with high activity only miss 7,918 to 29,433 variants each, and 12 distinct triplets of siRNAs can recognize each and every one of the > 13,000,000 sequenced variants (see Figure 5a).

Several ‘variant of concern’ lineages have emerged since the beginning of 2021 (*i.e*.: SARS-CoV-2 lineages exhibiting higher transmissibility, or more severe symptoms, or substantial escape from previously-acquired immunity, or limited response to existing treatments or diagnostic procedures). Importantly, and despite the > 18-month-long evolution of the virus in the human population, each of these variants of concern is efficiently recognized by the siRNAs that we designed in January2021 (see Figure 5b). This is essentially due to the fact that such variants tend to accumulate mutations in the *Spike* gene, whose protein product is exposed to the host immune system (hence, under apositive selection pressure) while our siRNAs target genes in Orf1ab (Candidates #2, 3 and 4 target the *Pol* gene, Candidates #5 and 8 target the *Nsp8* gene and Candidate 6 targets the *Hel* gene) (see Figures 4, 5c and Supplementary Figure S4).

## Discussion

Despite the prompt development of efficient and safe vaccines against SARS-CoV-2, the COVID-19 outbreak has claimed more than 6.5million human lives, and caused countless difficulties in the social and economic life. Several treatments have been developed, including anti-inflammatory medication for the most severely affected patients [64], polymerase inhibitors [65] and monoclonal antibodies [66] for nonhospitalized patients.

Besides small molecules and protein-based drugs, the development of siRNAs offers an attractive possibility, provided that suitable administration methods are developed. Biochemical determinants of their repressive activity are very precisely described, allowing efficient design of active siRNAs with limited predicted secondary effects. The great adaptability of siRNA design guarantees asimple and efficient update of siRNA sequences when novel viral variants emerge.

The rapid availability of many viral variant sequences has been amajor innovation during the COVID-19 outbreak, and any future viral epidemics is expected to be characterized at least as fast. Using such avariant sequence corpus as the only required input, and applying established rules in siRNA activity, we could design 8 siRNAs with apredicted high antiviral efficiency– with 6 of them indeed proving highly active *ex vivo*. These siRNAs were raised against highly-conserved regions of the SARS-CoV-2 genome as known in January2021. These regions were not only very constant among the ≈300,000 sequenced variants at that time– they also proved constant since then despite 18 more months of viral circulation in the human population, and our 6 highly active siRNAs still exhibit aperfect 13-mer match to almost every variant as of September2022– with 3-siRNA combinations being sufficient to recognize every correctly sequenced variant at that date. It is nonetheless expected that resistant viral variants would probably be selected if such siRNAs were widely administered to the human population. To reduce such risks, it would probably be necessary to track viral variant emergence and to update siRNA design periodically, always administering combinations of siRNAs targeting distinct viral regions (hence: unlikely to mutate in the same variant) rather than asingle siRNA.

The main obstacle to siRNA usage *invivo* lies in the difficulty of delivery to the target organs (in the case of COVID-19: respiratory epithelia). But the administration of anebulized siRNA solution by inhalation is under development [30] and it gave promising results with chemically-modified or unmodified siRNAs directed against SARS-CoV-2 [58,61]. Optimal timing for siRNA administration would also need to be optimized (*e.g*., administering the siRNAs at various time points before or after viral exposure, then scoring clinical outcomes), because the amplitude of viral replication and its anatonomical localization vary greatly in the course of COVID-19 infection [67]. If arobust method for siRNA administration can be optimized, siRNAs would therefore offer asimple, efficient and easily adaptable therapeutic response in case of future viral pandemics.

## Supplementary Material

Supplemental Material

## Data Availability

Raw, intermediate and processed data, as well as every script used for the analyses shown in the article, are accessible at https://github.com/HKeyHKey/Houbron_et_al_2023.

## References

[1] Ghildiyal M, Zamore PD. Small silencing RNAs: an expanding universe. Nat Rev Genet. 2009;10(2):94–108. 19148191 10.1038/nrg2504 PMC2724769

[2] Carmell MA, Xuan Z, Zhang MQ, etal. The Argonaute family: tentacles that reach into RNAi, developmental control, stem cell maintenance, and tumorigenesis. Genes Dev. 2002;16(21):2733–2742. DOI:10.1101/gad.1026102 12414724

[3] Elbashir SM, Lendeckel W, Tuschl T. RNA interference is mediated by 21- and 22-nucleotide RNAs. Genes Dev. 2001;15(2):188–200. 11157775 10.1101/gad.862301 PMC312613

[4] Elbashir SM, Martinez J, Patkaniowska A, etal. Functional anatomy of siRNAs for mediating efficient RNAi in *Drosophila melanogaster* embryo lysate. Embo J. 2001;20(23):6877–6888. DOI:10.1093/emboj/20.23.6877 11726523 PMC125328

[5] Llave C, Xie Z, Kasschau KD, etal. Cleavage of Scarecrow-like mRNA targets directed by aclass of *Arabidopsis* miRNA. Science. 2002;297(5589):2053–2056. DOI:10.1126/science.1076311 12242443

[6] Kasschau KD, Xie Z, Allen E, etal. P1/HC-Pro, aviral suppressor of RNA silencing, interferes with *Arabidopsis* development and miRNA function. Dev Cell. 2003;4(2):205–217. DOI:10.1016/S1534-5807(03)00025-X 12586064

[7] Becker WR, Ober-Reynolds B, Jouravleva K, etal. High-throughput analysis reveals rules for target RNA binding and cleavage by AGO2. Mol Cell. 2019;75(4):741–755. DOI:10.1016/j.molcel.2019.06.012 31324449 PMC6823844

[8] Holen T, Amarzguioui M, Wiiger MT, etal. Positional effects of short interfering RNAs targeting the human coagulation trigger tissue factor. Nucleic Acids Res. 2002;30(8):1757–1766. DOI:10.1093/nar/30.8.1757 11937629 PMC113209

[9] Amarzguioui M, Holen T, Babaie E, etal. Tolerance for mutations and chemical modifications in a siRNA. Nucleic Acids Res. 2003;31(2):589–595. DOI:10.1093/nar/gkg147 12527766 PMC140512

[10] Ding H, Schwarz DS, Keene A, etal. Selective silencing by RNAi of adominant allele that causes amyotrophic lateral sclerosis. Aging Cell. 2003;2(4):209–217. DOI:10.1046/j.1474-9728.2003.00054.x 12934714

[11] Haley B, Zamore PD. Kinetic analysis of the RNAi enzyme complex. Nat Struct Mol Biol. 2004;11(7):599–606. 15170178 10.1038/nsmb780

[12] Martinez J, Patkaniowska A, Urlaub H, etal. Single-stranded antisense siRNAs guide target RNA cleavage in RNAi. Cell. 2002;110(5):563–574. DOI:10.1016/S0092-8674(02)00908-X 12230974

[13] Meister G, Landthaler M, Patkaniowska A, etal. Human Argonaute2 mediates RNA cleavage targeted by miRNAs and siRNAs. Mol Cell. 2004;15(2):185–197. DOI:10.1016/j.molcel.2004.07.007 15260970

[14] Liu J, Carmell MA, Rivas FV, etal. Argonaute2 is the catalytic engine of mammalian RNAi. Science. 2004;305(5689):1437–1441. DOI:10.1126/science.1102513 15284456

[15] Rivas FV, Tolia NH, Song JJ, etal. Purified Argonaute2 and an siRNA form recombinant human RISC. Nat Struct Mol Biol. 2005;12(4):340–349. DOI:10.1038/nsmb918 15800637

[16] Park MS, Phan HD, Busch F, etal. Human Argonaute3 has slicer activity. Nucleic Acids Res. 2017;45(20):11867–11877. DOI:10.1093/nar/gkx916 29040713 PMC5714244

[17] Bartel DP. MicroRNAs: target recognition and regulatory functions. Cell. 2009;136(2):215–233. 19167326 10.1016/j.cell.2009.01.002 PMC3794896

[18] Hutvágner G, Zamore PD. AmicroRNA in a multiple-turnover RNAi enzyme complex. Science. 2002;297(5589):2056–2060. 12154197 10.1126/science.1073827

[19] Yekta S, Shih IH, Bartel DP. MicroRNA-directed cleavage of HOXB8 mRNA. Science. 2004;304(5670):594–596. 15105502 10.1126/science.1097434

[20] Davis E, Caiment F, Tordoir X, etal. RNAi-mediated allelic trans-interaction at the imprinted *Rtl1/Peg11* locus. Curr Biol. 2005;15(8):743–749. DOI:10.1016/j.cub.2005.02.060 15854907

[21] Shin C, Nam JW, Farh KK, etal. Expanding the microRNA targeting code: functional sites with centered pairing. Mol Cell. 2010;38(6):789–802. DOI:10.1016/j.molcel.2010.06.005 20620952 PMC2942757

[22] Karginov FV, Cheloufi S, Chong MM, etal. Diverse endonucleolytic cleavage sites in the mammalian transcriptome depend upon microRNAs, Drosha, and additional nucleases. Mol Cell. 2010;38(6):781–788. DOI:10.1016/j.molcel.2010.06.001 20620951 PMC2914474

[23] Iwakawa HO, Tomari Y. The functions of microRNAs: mRNA decay and translational repression. Trends Cell Biol. 2015;25(11):651–665. 26437588 10.1016/j.tcb.2015.07.011

[24] Silva JM, Hammond SM, Hannon GJ. RNA interference: apromising approach to antiviral therapy? Trends Mol Med. 2002;8(11):505–508. 12421679 10.1016/s1471-4914(02)02421-8

[25] Wood H. FDA approves patisiran to treat hereditary transthyretin amyloidosis. Nat Rev Neurol. 2018;14(10):570. 10.1038/s41582-018-0065-0 30158559

[26] Scott LJ. Givosiran: first approval. Drugs. 2020;80(3):335–339. 32034693 10.1007/s40265-020-01269-0

[27] Scott LJ, Keam SJ. Lumasiran: first approval. Drugs. 2021;81(2):277–282. 33405070 10.1007/s40265-020-01463-0

[28] Lamb YN. Inclisiran: first approval. Drugs. 2021;81(3):389–395. 33620677 10.1007/s40265-021-01473-6 PMC7900795

[29] Keam SJ. Vutrisiran: first approval. Drugs. 2022;82(13):1419–1425. 35997942 10.1007/s40265-022-01765-5

[30] Qiu Y, Lam JK, Leung SW, etal. Delivery of RNAi therapeutics to the airways— from bench to bedside. Molecules. 2016;21(9):1249. DOI:10.3390/molecules21091249 27657028 PMC6272875

[31] Wu F, Zhao S, Yu B, etal. Anew coronavirus associated with human respiratory disease in China. Nature. 2020;579(7798):265–269. DOI:10.1038/s41586-020-2008-3 32015508 PMC7094943

[32] Elbe S, Buckland-Merrett G. Data, disease and diplomacy: GISAID’s innovative contribution to global health. Glob Chall. 2017;1(1):33–46. 31565258 10.1002/gch2.1018 PMC6607375

[33] Bartel DP. Metazoan MicroRNAs. Cell. 2018;173(1):20–51. 29570994 10.1016/j.cell.2018.03.006 PMC6091663

[34] De N, Young L, Lau PW, etal. Highly complementary target RNAs promote release of guide RNAs from human Argonaute2. Mol Cell. 2013;50(3):344–355. DOI:10.1016/j.molcel.2013.04.001 23664376 PMC3746828

[35] Chable-Bessia C, Boull C, Neyret A, etal. Low selectivity indices of ivermectin and macrocyclic lactones on SARS-CoV-2 replication in vitro. COVID. 2022;2(1):60–75. DOI:10.3390/covid2010005

[36] Laplantine E, Chable-Bessia C, Oudin A, etal. The FDA-approved drug Auranofin has adual inhibitory effect on SARS-CoV-2 entry and NF-κBsignaling. iScience. 2022;25(10):105066. DOI:10.1016/j.isci.2022.105066 36093378 PMC9439859

[37] Ambike S, Cheng CC, Feuerherd M, etal. Targeting genomic SARS-CoV-2 RNA with siRNAs allows efficient inhibition of viral replication and spread. Nucleic Acids Res. 2022;50(1):333–349. DOI:10.1093/nar/gkab1248 34928377 PMC8754636

[38] Tafer H, Ameres SL, Obernosterer G, etal. The impact of target site accessibility on the design of effective siRNAs. Nat Biotechnol. 2008;26(5):578–583. DOI:10.1038/nbt1404 18438400

[39] Schwarz DS, Ding H, Kennington L, etal. Designing siRNA that distinguish between genes that differ by asingle nucleotide. PLoS Genet. 2006;2(9):e140. DOI:10.1371/journal.pgen.0020140 16965178 PMC1560399

[40] Wee LM, Flores-Jasso CF, Salomon WE, etal. Argonaute divides its RNA guide into domains with distinct functions and RNA-binding properties. Cell. 2012;151(5):1055–1067. DOI:10.1016/j.cell.2012.10.036 23178124 PMC3595543

[41] Schirle NT, Sheu-Gruttadauria J, MacRae IJ. Structural basis for microRNA targeting. Science. 2014;346(6209):608–613. 25359968 10.1126/science.1258040 PMC4313529

[42] Frank F, Sonenberg N, Nagar B. Structural basis for 5‘-nucleotide base-specific recognition of guide RNA by human AGO2. Nature. 2010;465(7299):818–822. 20505670 10.1038/nature09039

[43] Hu HY, Yan Z, Xu Y, etal. Sequence features associated with microRNA strand selection in humans and flies. BMC Genomics. 2009;10:413. 19732433 10.1186/1471-2164-10-413 PMC2751786

[44] Arvey A, Larsson E, Sander C, etal. Target mRNA abundance dilutes microRNA and siRNA activity. Mol Syst Biol. 2010;6(1):363. DOI:10.1038/msb.2010.24 20404830 PMC2872614

[45] Birmingham A, Anderson EM, Reynolds A, etal. 3‘ UTR seed matches, but not overall identity, are associated with RNAi off-targets. Nat Methods. 2006;3(3):199–204. DOI:10.1038/nmeth854 16489337

[46] Dang VT, Kassahn KS, Marcos AE, etal. Identification of human haploinsufficient genes and their genomic proximity to segmental duplications. Eur JHum Genet. 2008;16(11):1350–1357. DOI:10.1038/ejhg.2008.111 18523451

[47] Aza-Blanc P, Cooper CL, Wagner K, etal. Identification of modulators of TRAIL-induced apoptosis via RNAi-based phenotypic screening. Mol Cell. 2003;12(3):627–637. DOI:10.1016/S1097-2765(03)00348-4 14527409

[48] Schwarz DS, Hutvágner G, Du T, etal. Asymmetry in the assembly of the RNAi enzyme complex. Cell. 2003;115(2):199–208. DOI:10.1016/S0092-8674(03)00759-1 14567917

[49] Khvorova A, Reynolds A, Jayasena SD. Functional siRNAs and miRNAs exhibit strand bias. Cell. 2003;115(2):209–216. 14567918 10.1016/s0092-8674(03)00801-8

[50] Nykänen A, Haley B, Zamore PD. ATP requirements and small interfering RNA structure in the RNA interference pathway. Cell. 2001;107(3):309–321. 11701122 10.1016/s0092-8674(01)00547-5

[51] Harcourt J, Tamin A, Lu X, etal. Severe acute respiratory syndrome coronavirus 2 from patient with coronavirus disease, United States. Emerg Infect Dis. 2020;26(6):1266–1273. DOI:10.3201/eid2606.200516 32160149 PMC7258473

[52] Blanco-Melo D, Nilsson-Payant BE, Liu WC, etal. Imbalanced host response to SARS-CoV-2 drives development of COVID-19. Cell. 2020;181(5):1036–1045. DOI:10.1016/j.cell.2020.04.026 32416070 PMC7227586

[53] Khaitov M, Nikonova A, Shilovskiy I, etal. Silencing of SARS-CoV-2 with modified siRNA-peptide dendrimer formulation. Allergy. 2021;76(9):2840–2854. DOI:10.1111/all.14850 33837568 PMC8251148

[54] Idris A, Davis A, Supramaniam A, etal. A SARS-CoV-2 targeted siRNA-nanoparticle therapy for COVID-19. Mol Ther. 2021;29(7):2219–2226. DOI:10.1016/j.ymthe.2021.05.004 33992805 PMC8118699

[55] Tolksdorf B, Nie C, Niemeyer D, etal. Inhibition of SARS-CoV-2 replication by asmall interfering RNA targeting the leader sequence. Viruses. 2021;13(10):2030. DOI:10.3390/v13102030 34696460 PMC8539227

[56] Niktab I, Haghparast M, Beigi MH, etal. Design of advanced siRNA therapeutics for the treatment of COVID-19. Meta Gene. 2021;29:100910. 33996501 10.1016/j.mgene.2021.100910 PMC8106235

[57] Sohrab SS, El-Kafrawy SA, Azhar EI. In silico prediction and experimental evaluation of potential siRNAs against SARS-CoV-2 inhibition in Vero E6 cells. JKing Saud Univ Sci. 2022;34(4):102049. 35493709 10.1016/j.jksus.2022.102049 PMC9040457

[58] Yogev O, Weissbrod O, Battistoni G, etal. From a genome-wide screen of RNAi molecules against SARS-CoV-2 to avalidated broad-spectrum and potent prophylaxis. Commun Biol. 2023;6(1):277. DOI:10.1038/s42003-023-04589-5 36928598 PMC10019795

[59] Sohrab SS, El-Kafrawy SA, Azhar EI. Effect of insilico predicted and designed potential siRNAs on inhibition of SARS-CoV-2 in HEK-293 cells. JKing Saud Univ Sci. 2022;34(4):101965. 35313445 10.1016/j.jksus.2022.101965 PMC8925144

[60] Friedrich M, Pfeifer G, Binder S, etal. Selection and validation of siRNAs preventing uptake and replication of SARS-CoV-2. Front Bioeng Biotechnol. 2022;10:801870. 35309990 10.3389/fbioe.2022.801870 PMC8925020

[61] Chang YC, Yang CF, Chen YF, etal. AsiRNA targets and inhibits abroad range of SARS-CoV-2 infections including Delta variant. EMBO Mol Med. 2022;14(4):e15298. DOI:10.15252/emmm.202115298 35138028 PMC8988202

[62] Becker J, Stanifer ML, Leist SR, etal. Ex vivo and invivo suppression of SARS-CoV-2 with combinatorial AAV/RNAi expression vectors. Mol Ther. 2022;30(5):2005–2023. DOI:10.1016/j.ymthe.2022.01.024 35038579 PMC8758558

[63] Nabiabad HS, Amini M, Demirdas S. Specific delivering of RNAi using Spike’s aptamer-functionalized lipid nanoparticles for targeting SARS-CoV-2: astrong anti-Covid drug in aclinical case study. Chem Biol Drug Des. 2022;99(2):233–246. 34714580 10.1111/cbdd.13978 PMC8653378

[64] Horby P, Lim WS, Emberson JR, etal. Dexamethasone in hospitalized patients with Covid-19. NEngl JMed. 2021;384(8):693–704. 10.1056/NEJMoa2021436 PMC7383595 32678530

[65] Jayk Bernal A, Gomes da Silva MM, Musungaie DB, etal. Molnupiravir for oral treatment of Covid-19 in nonhospitalized patients. NEngl JMed. 2022;386(6):509–520. DOI:10.1056/NEJMoa2116044 PMC8693688 34914868

[66] Gottlieb RL, Nirula A, Chen P, etal. Effect of bamlanivimab as monotherapy or in combination with etesevimab on viral load in patients with mild to moderate COVID-19: arandomized clinical trial. JAMA. 2021;325(7):632–644. DOI:10.1001/jama.2021.0202 33475701 PMC7821080

[67] Rockx B, Kuiken T, Herfst S, etal. Comparative pathogenesis of COVID-19, MERS, and SARS in anonhuman primate model. Science. 2020;368(6494):1012–1015. DOI:10.1126/science.abb7314 32303590 PMC7164679

